# Introducing prediction intervals for sample means

**DOI:** 10.11613/BM.2024.030101

**Published:** 2024-08-05

**Authors:** Molly E. Contini, Jeffrey R. Spence, David J. Stanley

**Affiliations:** Department of Psychology, University of Guelph, Guelph, Canada

**Keywords:** prediction intervals, biostatistics, education, research methodology

## Abstract

Researchers and practitioners are typically familiar with descriptive statistics and statistical inference. However, outside of regression techniques, little attention may be given to questions around prediction. In the current paper, we introduce prediction intervals using fundamental concepts that are learned in descriptive and inferential statistical training (*i.e.,* sampling error, standard deviation). We walk through an example using simple hand calculations and reference a simple R package that can be used to calculate prediction intervals.

## Graphical abstract

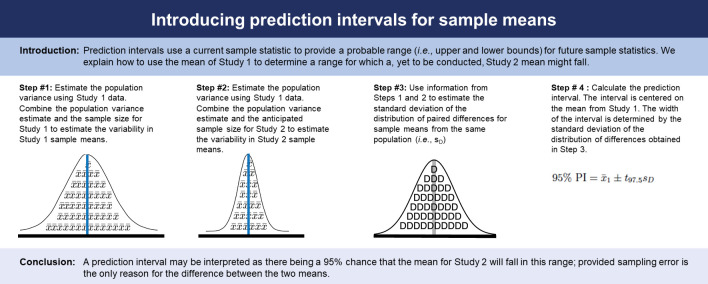

## Highlights

• Researchers and practitioners are typically familiar with descriptive statistics and statistical inference but may know little about prediction

• Prediction intervals use a current sample statistic to provide a range (*i.e.,* probabilistic upper and lower bounds) for future sample statistics

• In an applied example, we show how to calculate and interpret a prediction interval

• We use Study 1 sample data to generate a range of values that is likely to contain, a yet to be conducted, Study 2 mean

## Introduction and background

The prediction interval is a valuable but sometimes over-looked interval in statistical training (*1*-*4*). Many of the statistics familiar to researchers and practitioners are descriptive or inferential. Inferential statistics are primarily concerned with using sample data to estimate population attributes (*i.e.,* population parameters). In contrast, prediction intervals provide information about what result may be expected in a future sample (*5*-*7*). Prediction intervals use information from a past sample to estimate a range of values for a future sample statistic (*8*, *9*). In the current paper, we introduce readers to the logic underlying the calculation of prediction intervals. To ensure readers are able to easily follow the logic underlying these intervals we focus on the simplest case where we assume past and future samples are obtained from the same normally distributed population *via* simple random sampling.

A prediction interval for a sample mean is an interval that captures a future sample mean with a specified probability; 1-alpha (*9*). Prediction intervals model uncertainty in future data that can be expected due to sampling error. Sampling error is the difference between a sample estimate and the population parameter and will always be present when the sample size is smaller than the population. Correspondingly, sampling error decreases as sample size increases. In the case of prediction intervals, there are two sources of sampling variability that must be considered: (a) sampling variability associated with the current sample mean, and (b) the sampling variability associated with the future sample mean. As a result, prediction intervals incorporate sampling error from two estimates into a single interval.

For those interested in a more “mathematically precise definition of a prediction interval” we encourage readers to consult Hahn and Nelson’s more technical definitions of prediction intervals (*10*). Although we introduce prediction intervals focusing on the most straightforward scenario (sample means and normally distributed populations) it is also possible to construct prediction intervals in more complex scenarios. For example, prediction intervals are available for future observations and statistics such as effect sizes as well as for non-normal populations such as for exponential, binomial, Poisson, lognormal, and gamma distributions (*7*, *9*-*12*).

Because of their data-driven nature and future focus, prediction intervals are quite useful in a variety of research and applied contexts. Prediction intervals have been used in the context of multiple regression (*e.g.,* analyzing immunological responses to vaccinations); and have been recommended for use in meta-analyses examining treatment effectiveness (*13*, *14*). In laboratory medicine, prediction intervals have sometimes been used to establish reference ranges to interpret what test result may be considered normal or typical (*15*, *16*). Consequently, prediction intervals are an interesting statistic with both practical and research applications.

It is helpful to distinguish prediction intervals from confidence intervals because confidence intervals can sometimes incorrectly be interpreted as prediction intervals (*17*). For confidence intervals, the 95% value refers to the percentage of confidence intervals that are expected to contain the population parameter with repeated random sampling (*18*). In effect, a confidence interval is way of generating a plausible range of values for a population parameter. This interpretation differs sharply from that of a prediction interval which focuses on the generation of a plausible range of values for a future sample statistic. Consequently, confidence intervals do not perform well when used to capture future sample statistics. Simulation research has found that 95% confidence intervals capture future sample statistics at levels typically much lower than 95% and this reduced capture rate varies greatly across research scenarios (*7*).

In the sections to follow, we show how to calculate prediction intervals using sample-level estimates of a population parameter to generate a range of values that is likely to contain a subsequent sample-level estimate. We present an approach to understanding prediction intervals with the aim of making prediction intervals understandable and accessible to those with a background in descriptive and inferential statistics.

## An example

We begin by considering a fictitious example of a laboratory that tests for Analyte X which is normally distributed in the population of interest. As members of the laboratory, we are beginning an initiative to routinely screen for Analyte X concentrations in blood samples of people who live in a medium sized urban municipality. To begin, the laboratory randomly samples 100 adults (*i.e.*, n_1_ = 100) for inclusion in the first screening (*i.e.,* Study 1). Results of Study 1 reveal that the average concentration of Analyte X was 80 ppm (*x_people__1_* = 80). This sample mean is an estimate of the population mean (*i.e.,* the mean Analyte X concentrations for the population, the population of the municipality). This sample data can be used to generate an estimate of the population variance for Analyte X concentrations. In this example the estimated population variance is 225 (*i.e.,* s^2^_people1_ = 225). That is, 225 is an estimate of the variance of Analyte X concentrations for all adults in the municipality.

The laboratory plans to conduct follow-up testing six months later, we will call this future study, Study 2. Because of sampling error, the results from a new sample are expected to be different from the first sample. However, what results may be expected if nothing has changed in the environment to increase or decrease Analyte X concentrations? To get an estimate of what results can be expected, the lab can calculate a prediction interval. A prediction interval will provide the lab with a probable range of results that can be expected in test results simply due to sampling error. In the next sections, we walk through the components that go into calculating a prediction interval using this example.

## Statistical analysis

A prediction interval for sample means can be calculated using the *predictionInterval* package for *R* (*19*, *20*). R is a general purpose free open-source statistical software used by a range of research disciplines. Prediction intervals for means can be calculated in two simple ways: (a) using the statistical software with the *predictionInterval* package or (b) *via* a website app. Below, we present the code needed to calculate a prediction interval for sample means using the *predictionInterval* package (which assumes a normally distributed population). In the code, “M” is the mean of the sample data, “SD” is the sample estimate of the population standard deviation (*i.e.*, using *n*-1 in the denominator), “n” is the number of people in the current sample, and “rep.n” is the sample size of the future yet to be conducted study. The output indicates 95% prediction interval that ranges from 76.35 to 83.65.

library(predictionInterval)

pi.m(M = 80,

SD = sqrt(225),

n = 100,

rep.n = 200)

#Predictioninterval:

# 95% PI [76.35, 83.65].

## Prediction intervals and sampling distributions

**Figure 1:**
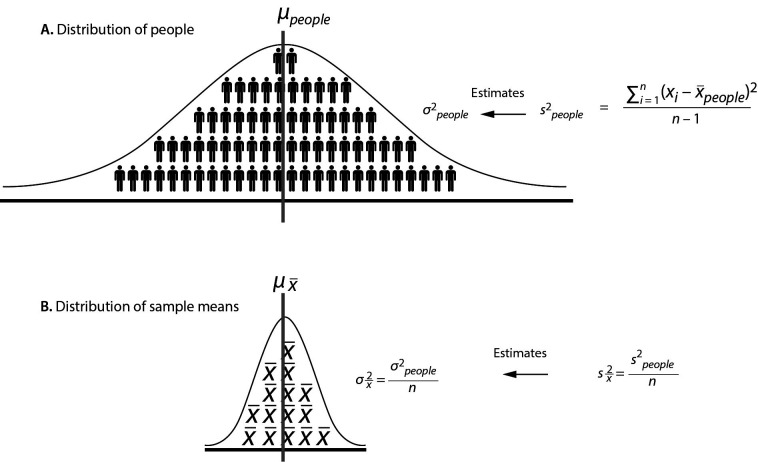
Population and sampling distribution of the mean.

A simple way to understand how prediction intervals work is to consider the laboratory’s Study 1 from a sampling perspective. To start, we can imagine that adults in the municipality vary in the amount of Analyte X concentrations in their blood. We can think of the variability in Analyte X concentrations for each person as a distribution. The distribution of Analyte X concentrations for all people in the municipality can be thought of as a population. We represent the Analyte X concentrations of the people forming the population using the notation μ*_people_* for the population mean and σ*^2^_people_* for the population variance (*i.e.,* the population parameters). The population-level variability in people’s Analyte X concentrations is illustrated in Figure 1A - which is depicted using people as icons as reminder individual Analyte X concentrations are represented in this figure. Notice that the population variance (σ*^2^_people_*) is unknown but estimated by *s^2^_people_* from Study 1.

Recall, the laboratory wants to determine what mean concentration of Analyte X is reasonable to expect in Study 2 based on the concentrations from Study 1. Said another way, the laboratory is interested in modeling how much a Study 2 mean, *x_people__2_*, is expected to differ from the Study 1 mean, *x_people__1_* due to sampling error. This difference can be expressed using the letter *D* as illustrated below (Equation (Eq.) 1):



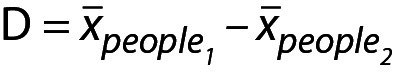



Mathematically modeling *D*, the difference between the Study 1 mean and the Study 2 mean, is a four-step process. First, we estimate the variance of the distribution for Study 1 means. Second, we estimate the variance of the distribution for the, yet to be conducted, Study 2 means. Third, we combine the information from Steps 1 and 2 to calculate the variance for the distribution of mean differences (*i.e.*, *D*). Finally, we calculate a prediction interval based on the standard deviation of the mean differences. These steps are outlined below.

## Step 1: Estimating sampling error in Study 1

We begin by creating a mathematical model for Study 1 results. That is, we imagine repeating Study 1 (using *n_1_ = 100*) an infinite number of times and calculating a mean for each study, resulting in an infinite set of sample means. This infinitely large set of sample means is collectively referred to as the sampling distribution of the mean. Thus, a sampling distribution of the mean in this context is a distribution of means calculated from all possible random samples of *n* = 100 from the population. This type of sampling distribution is illustrated in Figure 1B - the distribution is shaded with the sample mean symbol as a reminder it is a distribution that represents mean Analyte X concentrations not Analyte X concentrations for individuals.

In Figure 1B, we use the symbol *μ_x_* to refer to the mean of a distribution of sample means and the symbol to refer to the variance of this distribution of sample means. Collectively, the sampling distribution of means, in Figure 1B, represents all possible sample mean outcomes of Study 1, which use a sample size of 100. Even though the laboratory only collected one of these possible outcomes, the variance of this distribution is estimated from the sample data collected in Study 1. As depicted in Figure 1, to calculate this value, we do not actually need the infinitely large distribution of sample means to calculate its variance. The variance of this distribution of sample means (i.e.,) is linked to the variance of the distribution of individuals in the population (σ*^2^_people_*) by the sample size (n). This relation is presented in Eq. 2 below.



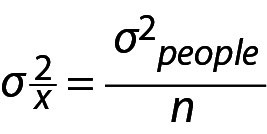



Because σ*^2^_people_* is a population parameter and is not typically known, we will need to use an estimate (*i.e.*, *s^2^_people_*) of the population-level variance from Study 1. To obtain *s^2^_people_*, we would use the formula for estimating the population-level variance from sample data, illustrated below. In this example, 225 is an estimate, based on sample data, of the variance in the Analyte X concentrations for all adults in the municipality (*i.e.,* the population).



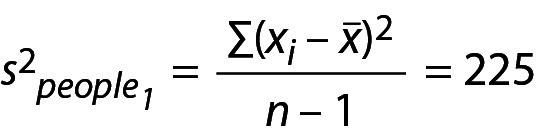



We can use an estimate of the population-level variance to obtain an estimate of the variance of the distribution of sample means, using Eq. 4 below



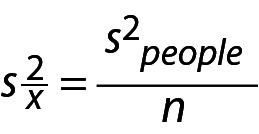



With this formula, we can estimate the variance of the distribution of sample means for Study 1 *via* the calculation below. Note, we now use the subscript 1 to identify that this is the estimate for Study 1,



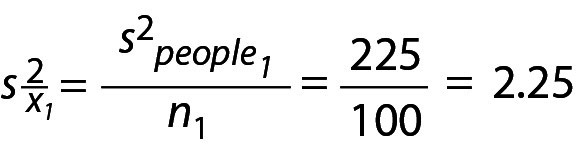



**Figure 2:**
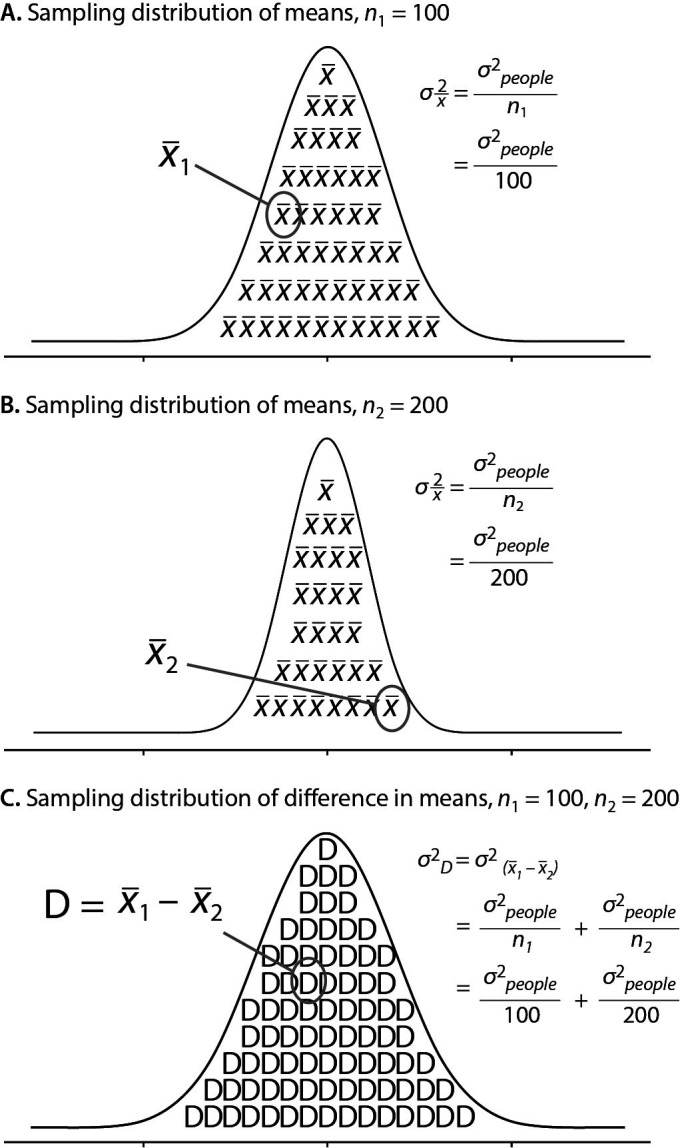
Sampling distributions with two different sample sizes.

A visual representation of this sampling distribution can be found in Figure 2A. In continuing with the goal of forecasting probable differences between the average Analyte X concentrations that might be found between Study 1 and Study 2, the laboratory also needs a model of the yet to be conducted Study 2. To do so, we can use the same technique to estimate the variance of the sampling distribution for Study 1 for, the yet to be conducted, Study 2, by using the data from Study 1.

## Step 2: Estimating sampling error in the future study (Study 2)

At first glance it may not appear possible to use the same technique from Study 1 for the yet to be conducted Study 2. This is because Study 2 is a hypothetical future study that has not been conducted. However, the calculation of a prediction interval does not require any data from Study 2 - we can use data from Study 1. Specifically, the formula for the variance of the sampling distribution of the mean for Study 2 requires an estimate of the population variance. Fortunately, we can use the estimate of the population variance from Study 1 for Study 2. Crucially, it is assumed that both samples used in Study 1 and Study 2 are from the same population, the municipality. Consequently, *s^2^_people1_* is an estimate of the population variance that is relevant to both Study 1 and Study 2 because both studies sample from the same population.

Consequently, the same rational that we used in Study 1 also applies to Study 2. The only thing that changes is the sample size estimate that the lab will use in Study 2. That is, we adapt Eq. 5, repeated below, by using the sample size of the second study, n_2_ = 200, in combination with the population-variance estimate from the first study, *s^2^_people1_*,



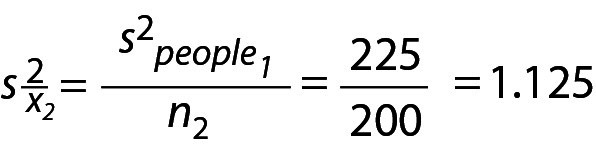



This equation uses the subscript 2 for as a reminder that it is an estimate of the variance of the sample means in Study 2. The resulting value, 1.125, is an estimate of the variability of all possible mean Analyte X concentrations for random samples of 200 in Study 2 - assuming these samples are drawn from the same population as Study 1. This sampling distribution variance for Study 2 is estimated entirely from Study 1 data (Study 2 data has not been collected) and is illustrated in Figure 2B.

Now the laboratory has estimates of the variance of the distribution of sample means for both Study 1 and, the yet to be conducted, Study 2. However, neither of the variance estimates are of direct interest. We merely use them as inputs in the calculation for the variance of the difference in sample means.

## Step 3: Estimating sampling error of the difference between means

In trying to set expectations for the second Analyte X sample mean, the laboratory is effectively interested in modeling how much the average Analyte X concentration from Study 2 is expected differ from the average Analyte X concentration from Study 1. Consequently, we need to model the mean difference between Study 1 and Study 2 results. This difference is expressed using the letter *D* see Eq. 7 below:



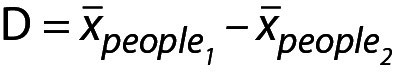



We need a model of how *D* will vary when both Study 1 and Study 2 are conducted a large number of times. In other words, we need to know how *D* is expected to vary across a large number of samples. To obtain the variance of the sampling distribution for *D*, we can use the variance sum law, see Eq. 8 below, which can be used to provide the variance for the difference (or sum) of independent variables:



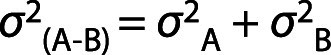



With the variance sum law, the laboratory can calculate the variance of a column of difference *(A - B)* using only knowledge of the variance of A and the variance of B. We can see an illustration of this in Table 1.

**Table 1:** Illustration of the variance sum law for two independent variables

**A**	**B**	**(A-B)**
σ^2^*_A_* = 12.64	σ^2^*_B_* = 2.00	σ^2^*_(A-B)_* = 14.64
9	3	6
2	4	-2
6	5	1
12	6	6
4	7	-3
Note: Variances were calculated using N in the denominator. Because A and B are independent COV(A,B) = 0.		

Using the variance sum law, we can determine the variance of the sampling distribution for *D* where *D* = *x_1_* – *x_2_*. This formula is illustrated below using the population-level notion from the previous two sections. Note the population variance, σ*^2^_people_*, is the same for both sample sizes (*i.e.,* for Study 1 and Study 2). The sampling distribution for the difference between the Study 1 and Study 2 means is illustrated in Figure 2C and Eq. 9



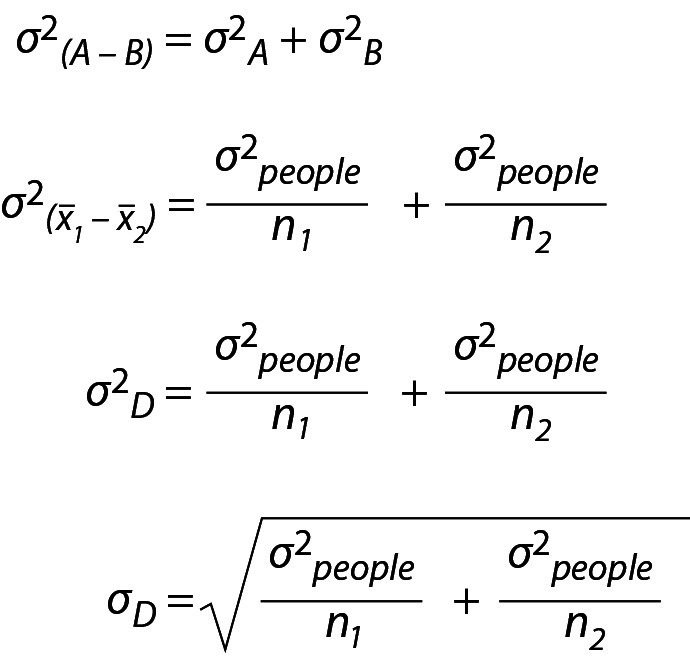



As before, because population values are unknown, we need to estimate the variance of the distribution of mean difference in sample means, see Figure 2C, using Eq. 10. Again, because Study 1 and Study 2 are samples from the same population we can use *s^2^_people1_* as the estimate of the population variance for both studies,



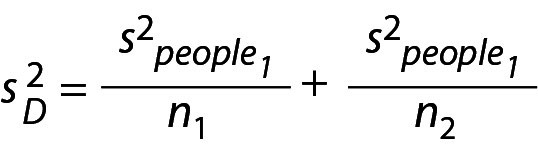



Inserting the previously calculated values we obtain (Eq. 11):



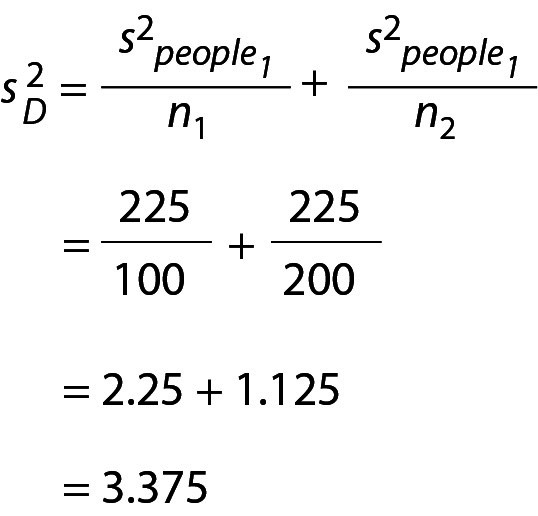



The corresponding estimated standard deviation of the distribution of mean differences is presented in Eq. 12



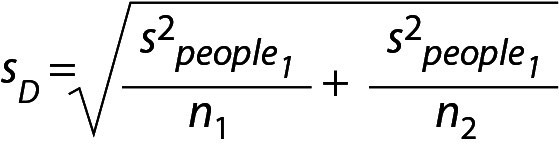



Consequently, the standard deviation for the distribution of mean differences is 1.837117. The corresponding sampling distribution is illustrated in Figure 2C (Eq. 13)



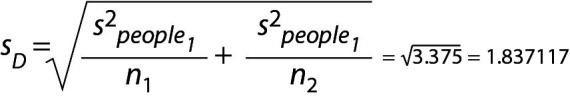



## Step 4: Calculating the prediction interval

We can now use the above values to calculate a prediction interval. A prediction interval is constructed much like a confidence interval - though interpreted quite differently. A confidence interval is based on the distribution of sample means (*i.e.*, *x*) whereas a prediction interval is based on the distribution of differences (*i.e.*, *D*) in sample means. Consequently, to construct a prediction interval, the laboratory will use the sampling distribution of the difference between means *D*, that we reviewed above.

To do so we use the following formula (Eq. 14):



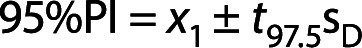



In Eq. 14 s*_D_* is the standard deviation of the sampling distribution for the difference between means (referred to by some as the standard error for the distribution differences in sample means). Here, the degrees of freedom for the *t*-distribution is based on Study 1 alone: *df* = n_1_ - 1 = 100 - 1 = 99.

Recall, in Study 1, *x_1_* = 80, *s^2^_people1_* = 225*_,_* and n_1_ = 100. Following from the sample size there are 99 degrees of freedom, *df* = 99. Using this information, we can calculate the prediction interval in the following way (Eq. 15):



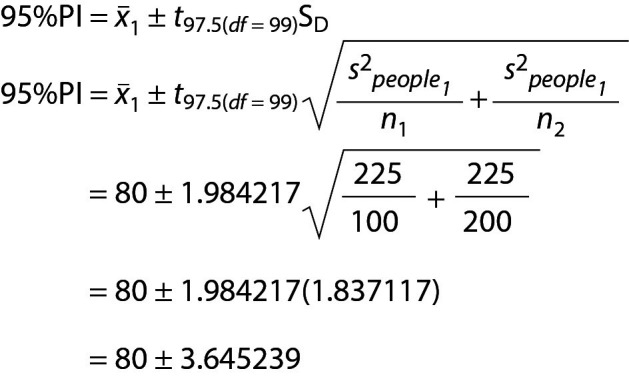



Consequently, the prediction interval is 95% PI [76.35, 83.65]. This can be interpreted as there being a 95% chance that the mean for Study 2 will fall in this range; provided sampling error is the only reason for the difference between the two means. Thus, the 95% prediction interval may be used to estimate a plausible range of values for the Study 2 mean. Consequently, although finding a mean outside of this range in Study 2 can be expected due to sampling error because it is a 95% prediction interval, not a 100% prediction interval, a mean outside of this range is unlikely and could be indicative of something other than sampling error occurring to cause the difference.

## Conclusions

In the current paper, we describe the prediction interval, a useful but sometimes overlooked interval (*1*-*4*). We reviewed prediction intervals for sample means obtained from normally distributed populations focusing on the logic underlying these calculations. Our aim was to make the prediction interval intuitive and accessible across a range of knowledge levels. We also provided information about an R package, *predictionInterval* that can easily calculate prediction intervals without any manual calculations (*19*).

There are a number of advantages to using prediction intervals. Prediction intervals are available for a variety of distributions and applications and they are relatively simple to calculate, even from small sample sizes. Moreover, a key assumption of prediction intervals is that the data have been collected *via* random sampling, thus if this assumption is violated, the intervals should be interpreted with caution. However, in the context of laboratory work where there is generally a high degree of control, the assumption of random sampling may be more readily satisfied compared to other contexts.

For additional practical examples on the use of prediction intervals interested readers may consult Coskun (*21*). This paper demonstrates how prediction intervals can be used in range of practical scenarios (*e.g.*, patient monitoring, evaluating laboratory instruments). Here, Coskun outlines a novel application of prediction intervals to generate personalized reference intervals such that repeated testing for a specific individual can be used to generate individual prediction intervals based on within-person variability (*21*). In earlier work, these intervals were referred to as personalized reference intervals (*22*). This approach provides a novel way to use prediction intervals to establish reference intervals. Others have argued against using prediction intervals to establish reference intervals (*23*).

Prediction intervals have some notable limitations. First, they assume the first sample and the second sample are obtained from the same population. Second, they assume random sampling was used to obtain the sample participants and only consider sampling error as a source of variability. For example, uncertainty due to measurement or lab errors is not taken into account when a prediction interval is calculated. The width of a prediction interval is determined entirely by random sampling error calculations.

Third, somewhat similar to significance testing and confidence intervals, the conclusions that can be drawn from prediction intervals may appear somewhat constrained and unsatisfactory. For example, even when there is a significant difference between two sample means you cannot be 100% certain there is a difference between the corresponding population means. Likewise, a 95% confidence interval may fail to capture a population mean; indeed, it will fail to do so 5% of time. Similarly, with prediction intervals, it is not possible to draw 100% definitive conclusions about results that fall either within or outside the interval. For example, a 95% prediction interval will fail to capture the next sample mean 5% of the time – due to random sampling error alone. However, there are other reasons that a sample mean could fall outside of the prediction interval. For example, if a researcher obtained a second sample mean from a different population (violating a prediction interval assumption) then the second sample mean might fall outside the prediction interval because the population has changed.

Confidence intervals are used to generate a plausible range of values for unknown population parameters; in contrast, prediction intervals are used to generate a plausible range of values for a future sample statistic. Both intervals are based on random sampling error calculations. Patel notes the past and the future distinction in his definition of a prediction interval as, “an interval which uses the results of a past sample to contain the results of a future sample from the same population with a specified probability” (*9*). Thus, a key difference between confidence intervals and prediction intervals is the fact that confidence intervals capture population parameters whereas prediction intervals capture a future sample statistic (*7*). Because prediction intervals model sampling error in two sources, (a) the past data and (b) the future data, they tend to be wider than confidence intervals. Confidence intervals can also sometimes be incorrectly interpreted as prediction intervals.

## Data Availability

No data was generated during this study, so data sharing statement is not applicable to this article.
